# Dynamic proteomic profiling of a unicellular cyanobacterium *Cyanothece *ATCC51142 across light-dark diurnal cycles

**DOI:** 10.1186/1752-0509-5-194

**Published:** 2011-12-01

**Authors:** Uma K Aryal, Jana Stöckel, Ravi K Krovvidi, Marina A Gritsenko, Matthew E Monroe, Ronald J Moore, David W Koppenaal, Richard D Smith, Himadri B Pakrasi, Jon M Jacobs

**Affiliations:** 1 Pacific Northwest National Laboratory, Richland, WA 99352, USA; 2 Department of Biology, Washington University, St. Louis, MO 63130, USA

## Abstract

**Background:**

Unicellular cyanobacteria of the genus *Cyanothece *are recognized for their ability to execute nitrogen (N_2_)-fixation in the dark and photosynthesis in the light. An understanding of these mechanistic processes in an integrated systems context should provide insights into how *Cyanothece *might be optimized for specialized environments and/or industrial purposes. Systems-wide dynamic proteomic profiling with mass spectrometry (MS) analysis should reveal fundamental insights into the control and regulation of these functions.

**Results:**

To expand upon the current knowledge of protein expression patterns in *Cyanothece *ATCC51142, we performed quantitative proteomic analysis using partial ("unsaturated") metabolic labeling and high mass accuracy LC-MS analysis. This dynamic proteomic profiling identified 721 actively synthesized proteins with significant temporal changes in expression throughout the light-dark cycles, of which 425 proteins matched with previously characterized cycling transcripts. The remaining 296 proteins contained a cluster of proteins uniquely involved in DNA replication and repair, protein degradation, tRNA synthesis and modification, transport and binding, and regulatory functions. Functional classification of labeled proteins suggested that proteins involved in respiration and glycogen metabolism showed increased expression in the dark cycle together with nitrogenase, suggesting that N_2_-fixation is mediated by higher respiration and glycogen metabolism. Results indicated that *Cyanothece *ATCC51142 might utilize alternative pathways for carbon (C) and nitrogen (N) acquisition, particularly, aspartic acid and glutamate as substrates of C and N, respectively. Utilization of phosphoketolase (PHK) pathway for the conversion of xylulose-5P to pyruvate and acetyl-P likely constitutes an alternative strategy to compensate higher ATP and NADPH demand.

**Conclusion:**

This study provides a deeper systems level insight into how *Cyanothece *ATCC51142 modulates cellular functions to accommodate photosynthesis and N_2_-fixation within the single cell.

## Background

Oxygenic photosynthetic cyanobacteria are widely recognized for their important role in the global carbon cycle [1], and have generated significant interest as a potential solution for carbon-neutral energy production and carbon (C) sequestration [2]. Some unicellular cyanobacteria such as *Cyanothece *and *Crocosphaera *are also capable of biological N_2_-fixation (diazotrophic) [3], and play a significant role in marine nitrogen (N) cycle [4]. One of the most important metabolic considerations for these organisms is that the enzyme nitrogenase, which is responsible for N_2_-fixation, is rapidly and irreversibly inactivated upon exposure to O_2_, a product of photosynthesis [5]. To overcome this, *Cyanothece *have developed mechanisms to switch/alternate the cellular and molecular machinery twice daily to temporally separate N_2_-fixation in the dark from photosynthesis in the light [6,7]. Components of this temporal segregation of incompatible metabolic functions are controlled by circadian rhythms and/or daily oscillations of bio-chemicals in response to the light-dark cycle [8].

Cyanobacteria utilize other molecular mechanisms to coordinate the distinct biochemical pathways associated with the acquisition and assimilation of C or N. For example, the PII protein (*glnB *homolog) regulates C and N nutrients in the cell by sensing the C/N ratio whereas thioredoxin (Trx) is involved in the light dependent regulation of many enzymes in photosynthetic electron transfer chain [9]. Similarly, the global transcriptional regulator NtcA, regulates cellular nitrogen by sensing the level of 2-oxoglutarate (2-OG) [10]. Thus, cellular metabolism in various cyanobacteria is intricately linked to and modified by growth conditions that include lighting regimes [11].

In *Cyanothece*, the removal of light triggers cascades of gene expression and protein synthesis changes that lead to a functional changeover from photosynthesis to N_2_-fixation [6,7]. Under lighted conditions the reverse occurs. In tandem with these processes there are hosts of other regulated activities that occur based upon an internal clock (circadian rhythm), rather than reacting to changing lighting conditions. This combination of response and anticipation of the light-dark cycle is rooted in the genome, and discerning these mechanisms would provide further insights into how cells synchronize biological functions with their daily light-dark cycles. Transcriptional regulation of photosynthesis and N_2_-fixation in *Cyanothece *and other cyanobacteria has been described previously [12-15], however relatively little is known of how these transcript changes actually affect protein concentrations. Recently, Himadri and co-workers [16] used label free static quantification to identify 250 proteins which showed oscillations between the light-dark cycles and of these 175 had corresponding transcript changes [12]. However, that was not a comprehensive study and we reasoned that the link between the transcriptomic and proteomic observations could be more efficiently addressed by the measurement of real-time protein expression changes using metabolic labeling with high mass accuracy capillary liquid chromatography (LC) mass spectrometry (MS) analysis.

Metabolic labeling of proteins using stable isotope labeling with amino acids in cell culture (SILAC) [17] has become a powerful technique for quantitative MS-based proteomics [18]. Traditionally, SILAC relies on metabolic incorporation of a given "light" (unlabeled) or "heavy" (labeled) form of the amino acid into the proteins, and the relative protein expression changes can be accurately measured by comparing the abundances of the light and heavy peptides [19]. While majority of the dynamic proteomic analysis using SILAC have used complete (saturated) labeling [20-23], principles of SILAC with incomplete (unsaturated) labeling with short labeling windows can also be useful to measure the dynamics of the protein turnover [24].

Recently, we defined a novel experimental approach to capture the dynamics of *Cyanothece *ATCC51142 proteome by using partial metabolic labeling with MS [25]. In this method, following culture growth in unlabeled medium for several days, cells were labeled for different time periods by adding heavy labeled isotope. Labeled amino acid was incorporated into all newly synthesized proteins, and the level of label incorporation at any time indicated the amount of newly synthesized proteins. As cells were partially labeled, each extractable protein contained both labeled and un-labeled amino acid, and their relative abundances provided the amount of new protein synthesis. Using this approach, we previously measured the temporal changes of protein expression in *Cyanothece *ATCC51142 under high light exposure [25]. In the present study, we sought to expand this technique for systems-wide dynamic proteomic profiling in *Cyanothece *ATCC51142 in diel regimes. Such study has the potential to reveal previously unknown clues of daily modulation of cellular functions and enhance our understanding of the link between the transcriptomic and proteomic data. This information is increasingly important as systems biology aims to integrate multiple 'omics' measurements.

Thus, the overall aim was to quantitatively monitor the light-dark specific changes in protein expression in real-time in *Cyanothece *ATCC 51142. This was performed by the time course measurement of heavy isotope incorporation into proteins over two consecutive 24-h light-dark diurnal cycles. Using this strategy in combination with high mass accuracy LC-MS/MS, we quantitatively measured 721 proteins with changing levels of isotope incorporation, which largely contained proteins involved in central metabolic processes including those involved to compartmentalize metabolic products and store energy, features that make *Cyanothece *ATCC51142 an ideal system for biofuel research. Proteins involved in CO_2_- and N_2_-fixation were expressed with different temporal profiles. In-depth analysis of these labeled proteins identified several previously unknown biochemical pathways that might be responsible for synchronizing the cellular metabolism within the daily light-dark cycles.

## Results and Discussions

### Qualitative and quantitative profiling of the proteome

**Figure 1:**
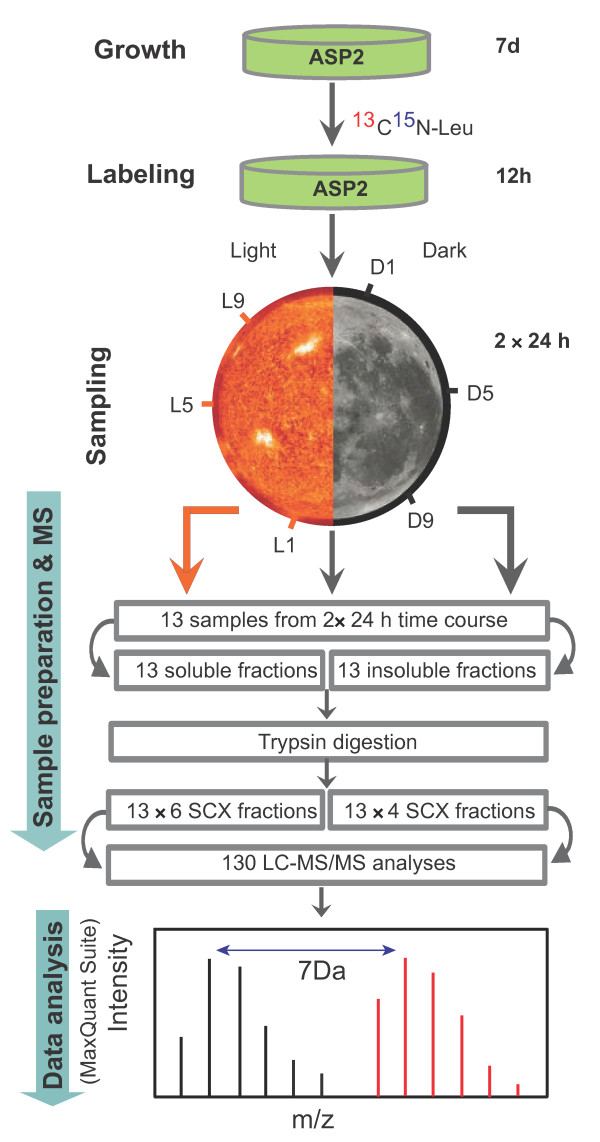
**Experimental workflow for metabolic labeling and identification of labeled peptides**. Cell lysate was initially divided into soluble and insoluble fractions and digested with trypsin. After desalting, tryptic peptides were SCX fractionated and subjected to high mass accuracy LC-MS/MS analysis. Details of these steps have been described in the experimental section.

**Figure 2:**
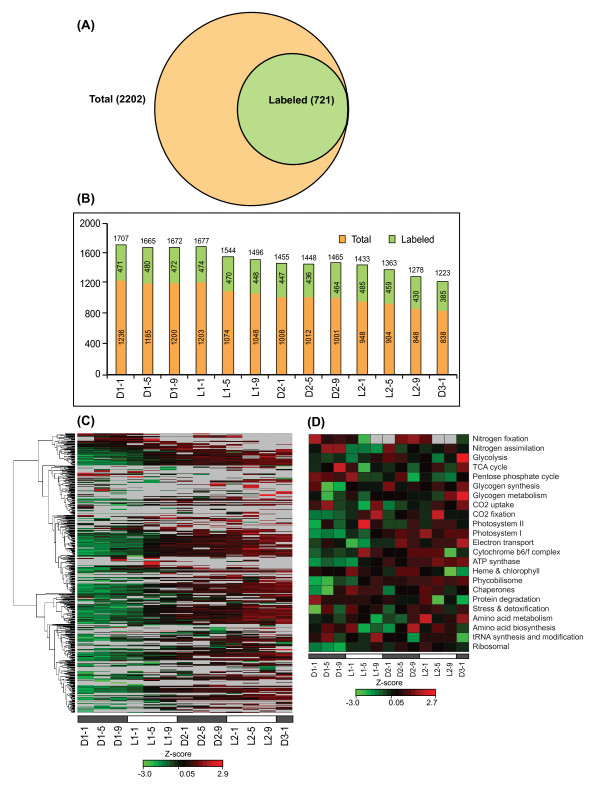
**Distribution of the labeled proteins across time points**. (A), Venn diagram summarizing the number of labeled proteins in the total identifications (labeled + unlabeled). The total 2202 proteins identified in this study are the leucine containing proteins only. (B), Distribution of the labeled proteins over the time course analysis. The numbers on the top of each bar show the total proteins (labeled + unlabeled), the green bars show the labeled proteins and the orange bars show proteins without labels. (C), Heatmap showing the changes in the abundances of the labeled proteins over time. The observed heavy (H) and light (L) peptide intensities were used to calculate RIA [I_H_/(I_H_+I_L_)], which were then exported to DanTE [59] for log_2 _based normalization and rolling into proteins using Reference Peptide based scaling (RRollup). The normalized RIA values were used for hierarchical clustering into the heatmap. (D), Functional classification of the labeled proteins based on KEGG pathway [27] and Gene Ontology (GO) analysis. The RIA for each category was calculated as an average of all the proteins belonging to each category across all time-course. The log2 transformed RIA values of all the labeled proteins can be found in Additional file 1, Table S2. The list of labeled peptides with their actual RIA is provided in the Additional file 1, Table S2.

*Cyanothece *have been shown to store excess C and N in the form of glycogen and cyanophycin granules and to utilize these compounds during starvation [11]. Our experimental strategy involved growing cultures under the diel regime (12 h L: 12 h D) in ASP_2 _medium without NaNO_3 _(nitrogen starvation) for seven days prior to adding the labeled leucine. (Figure 1). Sampling of partially-labeled *Cyanothece *ATCC51142 cells was initiated thirteen hours after the addition of the labeled leucine, and samples were collected at 4 h intervals across 13 time points for a time-course analysis (Figure 1). Extractable proteins were divided into soluble and insoluble fractions for each collection [16] and subjected to high resolution LC-MS/MS analysis after tryptic digestion and SCX separation (Figure 1). Allowing < 5 ppm average mass deviations and 1% FDR on both peptide and protein levels, we were able to identify 19327 leucine-containing peptides irrespective of label information, which mapped to 2202 proteins with at least two peptides per protein, representing 47% of the entire predicted proteome (Figure 2A).

In cyanobacteria, the synchronization of cellular functions with appropriate phases of the diurnal cycle must originate, in part, from the dynamic changes in protein expression, which is the net result of the contribution from a new protein synthesis in competition with protein degradation at any given time [26]. However, under our experimental strategy, the rates of degradation were assumed to be identical due to the fact that these versions are chemically indistinguishable molecules, and are indeed inseparable for degradation. As a consequence, changes in the abundance of a specific protein can be attributed due to changes in new protein synthesis which can be determined by the measure of label incorporation. Accordingly, accumulation of the label into proteins would be proportional to the rate at which they are synthesized, and degraded. Under our experimental set-up, all newly synthesized proteins would increase in label through the time of the experiment, but at different rates. So, proteins with rapid turnover accumulate label much faster than the low turnover proteins. We used Relative Isotope Abundances (RIA) [21,22,24] to monitor the temporal changes in protein expression across time points. RIA provides a measure of the fraction of the labeled peptides in the total (labeled + unlabeled) peptide pool for a particular protein, and this value can be used to determine actual new protein synthesis at any given time point. Achieving a complete (saturated) labeling (RIA ~1) as assumed for pooled classical SILAC based measurements, was not the goal of this study, however, the concept of partial (incomplete) labeling with short labeling windows (dynamic metabolic labeling) can be a powerful approach to monitor and visualize the proteome dynamics. In incomplete labeling, each protein contains both labeled and unlabeled amino acid, and its tryptic digestion generates both labeled and unlabeled peptides. At each time point, proteins were identified, and the changes in the partition of peptide ion current between the labeled and unlabeled peptides in LC-MS measured by RIA provide information on the real-time protein synthesis. For example, with an RIA of 0.55 at D1, NifH (*cce_0559*) has at least 55% of this protein newly synthesized over the preceding 13 hours at that time point (Additional file 1, Table S2). However, as proteins can be synthesized from amino acids (i. e. leucine) that can be derived by *de-novo *synthesis, these measured values likely represent the lower limit of the true turnover [18].

Using this procedure, we identified 2837 individual peptides with the pair-wise information for both the heavy and corresponding natural light version of the peptides (Additional file 2, Table S1) which mapped to 721 proteins (13.8% of the predicted proteome) (Figure 2A and Additional file 1, Table S2). Figure 2B shows the distribution of these labeled proteins compared with the total protein observations (labeled + unlabeled) whereas the distribution of the labeled peptides and their corresponding proteins across all time points are shown in Additional File 3, Figure S1A. Even though the analysis was performed at a uniform peptide amount across all samples, we saw a distinct decreasing trend of total identifications across the entire time course (Figure 2B), which is more obvious during the light phases. This downward trend appeared to be predominately influenced by the decrease in unlabeled protein identifications over time.

The differentially labeled proteins with their individual patterns of RIA across the time-course were subjected to hierarchical clustering generating the dendrogram as represented in Figure 2C. These data show a clear increase in the incorporation of metabolic label per protein over time (green to red), suggesting that isotope incorporation is driven by the cell growth. However, the proportion of the labeled amino acid varied among the individual proteins (RIA values) suggesting that isotope incorporation is also dictated by intracellular protein turnover.

Functional categorization of the labeled proteome from KEGG pathway [27], and Gene Ontology (GO) analysis showed that the actively synthesized labeled proteins belong to diverse individual biochemical pathways such as CO_2 _fixation and assimilation, N_2_-fixation and N assimilation, glycogen synthesis, glycogen metabolism, glycolysis and oxidative PP pathways (Figure 2D), and that many of these were maximally expressed at different phases of the light-dark cycle. For example, the majority of proteins involved in glycolysis, TCA cycle and PP cycle usually showed higher expression in the dark whereas those involved in CO_2 _fixation, heme, chlorophyll and phycobilins biosynthesis showed higher expression in the light cycle (Figure 2D). Again, proteins involved in N_2_-fixation, protein degradation, glycogen synthesis and metabolism, heme and chlorophyll biosynthesis showed relatively higher levels of isotope incorporation (black or red on the heat map) as compared with other functional categories. Interestingly, a total of 182 (25%) proteins with unknown functions were differentially labeled in between the light-dark cycle suggesting their potential roles in cellular metabolism, and highlight the importance of systematic characterization of their functions for better understanding of the physiology and cellular metabolism of *Cyanothece*.

### Comparison between transcriptional and proteomic data

**Figure 3:**
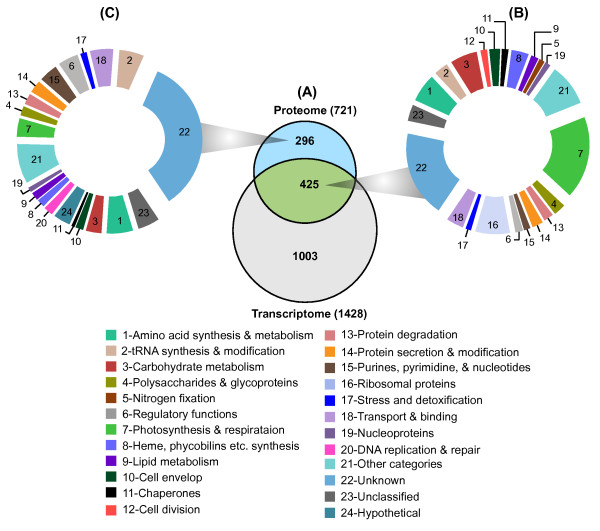
**Comparison between proteomic and previously obtained transcriptomic data sets**. (A), Venn diagram showing a panel of 721 labeled proteins and 1428 cycling transcripts [12]. In total, 425 labeled proteins matched with corresponding cycling transcripts (Green), which were categorized into different functional categories in (B). The non-overlapping 296 proteins (blue) were also classified into functional groups as represented in (C). Functional categorization in (B) and (C) based on KEGG pathway [27], and Gene Ontology (GO) analysis are numbered and clearly showed apparent differences indicating that different groups of proteins are regulated differently. The functional categories are labeled numbers.

Details of the transcriptional regulations of biological processes in *Cyanothece *and other unicellular cyanobacteria have been described in previous studies [12-15,28,29]. A robust approach to determine both cycling *Cyanothece *ATCC51142 transcripts and cycling proteins from near identical experiments has been previously reported [12,16] which data was used in the current study for comparison. Indeed, a major focus of the current study was to investigate exactly how these cycling transcripts/proteins would be reflected when viewed through the real time metabolic synthesis of proteins. There was no additional cycling analysis performed on the current dataset, other than to map the detected proteins onto the previously published and characterized data. Although transcriptional and translational events are generally correlated, concordances and discordances exist [30]. Previously [16], we identified 250 proteins oscillating between light-dark cycles, of which 175 matched to the cycling genes [12]. The oscillating proteins were determined based on Fourier score [29], fold change (≥ 1.5) between spectral counts and computed P-value of < 5% using two sample t-test [16]. Of the 721 labeled proteins identified in this study, 425 proteins (58.9%) matched with the previously characterized cycling transcripts (Figure 3). Similarly, out of the 250 oscillating proteins identified from label free approach [16], 145 matched with the labeled proteins (Additional file 3, Figure S1B). This protein subset represents those under active synthesis and having correspondence to well-defined cyclic transcripts. Of potential interest are the 296 (41.1%) actively synthesized proteins having no correspondence to cycling transcripts, and possibly represent those that are likely regulated at a post-transcriptional (translational or post-translational) level. For example, circadian clock protein KaiC1 (cce_0422), which rhythmically phosphorylates and dephosphorylates *in-vivo *and serves as a timing cue for assembly/disassembly of other circadian clock proteins such as KaiA and KaiB [31], was actively synthesized in the dark cycle but had no distinct cycling transcript (Additional file 1, Table S2) [12]. Functional classifications showed that large proportions of the labeled proteins were of unknown function in both overlapping and non-overlapping sub-sets. Among the known functions, photosynthesis, respiration, CO_2_-fixation, N_2_- fixation, carbohydrate metabolism, cell division and protein translation showed higher representation in the sub-set that overlap with the cycling transcripts whereas DNA replication and repairs, transport and binding, nucleotides metabolisms, and tRNA synthesis and modification, and regulatory functions showed higher representation in the non-overlapped sub-set. Interestingly, all the 32 ribosomal proteins identified in this study matched with the corresponding cycling transcripts [12]. Such proteins are obviously integral to the ribosome and are involved in the translation of transcripts encoded in cellular genomes, and may exert a level of developmental control over cell/tissue fate and function [32]. Thus, major differences of proteins for various functional classes were apparent indicating that diverse groups of proteins are regulated differently.

## Correlating dynamic expression of proteins and cellular functions

### Nitrogen fixation and assimilation

**Figure 4:**
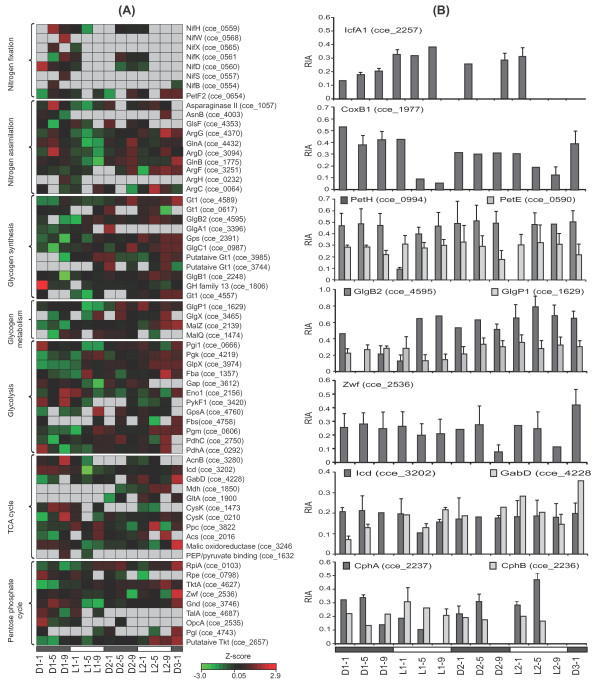
**Comparison of dynamic proteomic changes across time points**. (A), Heatmap showing changes in protein abundances of different functional categories over time. Heatmaps were generated as described in Figure 2B. The protein symbols (or abbreviated protein names) with ORF are shown on the right and the name of the functional group on the left. (B), Examples of proteins from different functional categories with the average RIA values of peptides (± SD) matching to that proteins, and showing the dynamic changes in expression patterns between light-dark cycles. Gt1, glycosyltransferase group 1; Fba, fructose-1, 6-bisphosphate aldolase. The heat maps of the remaining labeled proteins with their functional groups are shown in Additional file 4, Figure S2.

Expression of nitrogen fixation proteins NifH, D, and K were strongly cyclic across both diurnal cycles and limited to the dark cycle [16] (Figure 4A, first subset), with an actual loss of detection within the light compartment. NifB (cce_0554), a sensitive target for assessing the regulation of N_2_-fixation [15], was also abundant in the dark whereas NifW (cce_0568), a small protein with 116 residues (13.3 kDa) located in the 35 nitrogenase gene clusters, was abundant near the end of the first dark transition, but not observed in the second dark phase. This was also true for NifX (cce_0565) and NifS (0557). Interestingly, out of the 8 labeled domain of unknown function (DUF) proteins, DUF269 (cce_0566) was distinct, showing cycling expression with higher incorporation in the dark (Additional file 1, Table S2), in agreement with transcript data [12]. Although, the biological function of DUF269 is not clear, its expression pattern and genetic location within the 35 *nif *gene cluster clearly suggests a role in N_2_-fixation.

Under nitrogen limitation, cyanobacteria preferentially utilize alternative N sources [33]. GlnA (cce_4432) and GlsF (cce_4353) involved in nitrogen assimilation had increased synthesis in the dark (Figure 4A, second subset). Mechanistically, ammonium (NH_4_^+^) in the cell is trapped by GlnA [34] and converted to glutamate via GS (GlnA)-GOGAT (GlsF) cycle, and incorporated into other amino acids through the action of aminotransferase or transaminase [35]. GlnB (cce_1775), which activates the transcription of many nitrogen regulated genes by regulating kinase and phosphatase activities [36] also showed increased synthesis in the dark (Figure 4A, second subset). On the other hand, proteins involved in amino acid biosynthesis such as chorismate synthase; AroC (cce_4200) and acetolactate synthase large subunit; IlvB (cce_4227) showed higher synthesis during the light cycle (Additional file 1, Table S2). AroC catalyzes chorismate production, which is a key intermediate of phenylalanine, tyrosine, and tryptophan biosynthesis whereas IlvB catalyzes leucine, isoleucine and valine biosynthesis. Differential labeling of these proteins provide evidences of their active synthesis and role in diurnal growth.

### Photosynthesis and respiration

Carbon fixation machinery requires proper assembly and functioning of carboxysome [1]. Our data showed higher expression of IcfA1 (cce_2257) (Figure 4B, top panel) and bicarbontate transport system substrate-binding protein, CmpA (cce_0305) in the light cycle, and sodium dependent bicarbonate transporter; SbtA (cce_2939) in the dark cycle (Additional file 4, Figure S2). CmpA is highly homologous to the nitrate transport protein NrtA, and participates in the co-transport of bicarbonate, Ca^2+ ^and Na^+ ^or that Ca^2+ ^and Na^+ ^act as a cofactor in bicarbonate transport [37]. Glycolate oxidase (cce_3708), a key enzyme to convert 2-phosphoglycolate to PG and O_2 _[38] showed higher amount of label incorporation in the light cycle (Additional file 1, Table S2). 2-Phosphoglycolate is produced by the oxidase activity of rubisco when the O_2_-CO_2 _ratio increases. Excitation of PSII during the light period is expected to increase the O_2_-CO_2 _ratio and expression of glycolate oxidase may constitute an important cellular adaptation under the light period [39]. So, higher synthesis of several carboxysome proteins (RbcSL, IcfA1, CcmM, CcmK1, CcmK2, and CcmK4) together with ParA (cce_2448) and RbcR (cce_3731) in the light cycle might be an indication of active carboxysome functioning under the light period (Additional file 4, Figure S2 and Additional file 1, Table S2). In *Synechococcus elongatus *PCC 7942, a parA-like gene (*Synpcc7942_1833*) is known to control carboxysome organization [1], and ParA protein was also actively synthesized in the light cycle for *Cyanothece *ATCC51142, providing clues of cellular processes that control carboxysome assembly and function.

Expressions of PSI and PSII proteins were not strongly coupled with the light-dark cycles (Additional file 4, Figure S2). In particular, in PSII, Psb28 (cce_1599), PsbE (cce_1307) and PsbA3 (cce_0267) were more abundant in the dark while other PSII proteins (PsbD1, PsbC, Psb28-2, PsbO, Psaf, PsbP) were more abundant in the light cycle. This disparity might be a result of continuous assembly and disassembly of PSI and PSII proteins due to frequent light-mediated damage under normal cellular function. Phycobiliproteins, which play an important role for light harvesting and distributing energy to PSI and PSII, usually had higher levels of synthesis in the light phase (Additional file 4, Figure S2). In contrast, CoxB1 (cce_1977), a subunit of the terminal oxidase in the respiratory transport chain was predominant in the dark cycle (Figure 4B, second panel). Respiration has an important role in diazotrophy in cyanobacteria [7,40], and CoxB1 is likely to play a role in scavenging free O_2 _in the cytoplasm to prevent nitrogenase deactivation [7]. The transcripts of *coxA *were shown to increase in the dark cycle both under N_2_-suffcient and N_2_-deficient conditions [41]. Interestingly, soluble electron carrier ferredoxin-NADP oxidoreductase, PetH (cce_0994) showed increased synthesis in the dark cycle whereas plastocyanin PetE (cce_0590) showed increased synthesis in the light cycle (Figure 4B, third panel) suggesting that PetH might be involved in the respiratory and PetE in the photosynthesis electron transport.

### Central intermediary metabolism

Efficient cellular metabolism relies on the compartmentalization of enzymatic reactions [1]. In cyanobacteria, formation of glycogen during photosynthesis and their metabolism during N_2_-fixation is well known [7]. Consistent with this, synthesis of glycogen synthase; GlgA1 (cce_3396) peaked at the first light cycle (L1-3) with no detection at later time points (Figure 4A, third subset). Similarly, synthesis of 1, 4-glucan debranching enzyme; GlgB2 (cce_4595), which is involved in converting glucose to glycogen also peaked in the first light cycle and remained consistently high in the second light cycle (Figure 4B, 4th panel). In contrast, glycogen phosphorylase; GlgP1 (cce_1629) usually had higher levels of synthesis in the dark compared to the light cycle, suggesting higher rate of glycogen metabolism during N_2_-fixation (Figure 4B, 4th panel) [7,16]. Overall, we observed active synthesis of the majority of glycolysis and PP pathway enzymes at varied time points during the dark cycle (Figure 4A). Glucose 6-phosphate dehydrogenase, Zwf (cce_2536) redirects C flow and initiates the oxidation of glucose concomitantly to sugars and reducing equivalents in the form of NADPH, and represents the essential catabolic route for providing reductant for N_2_-fixation and respiration in *Cyanothece *ATCC51142 [12,16]. There was a slight trend in the synthesis rate of Zwf (Figure 4B, 5th panel) being increased in the dark, however there is no significant different in abundances between light-dark cycles. In cyanobacteria, the TCA cycle is incomplete [42]. Isocitrate dehydrogenase (Icd) converts isocitrate to 2-OG, which is utilized for the biosynthesis of glutamate via GS-GOGAT cycle [43]. Concurrently, we observed a slight trend in the synthesis of Icd (cce_3202) being increased in the dark and GabD, another enzyme known to participate in glutamate metabolism for the conversion of 2-OG to succinate [44] in the light cycle (Figure 4B, 6th panel), and might suggest that cells utilize different substrates as C and N sources. Increased expression of *icd *transcripts under N_2_-fixing conditions has been observed in other cyanobacteria [43].

**Figure 5:**
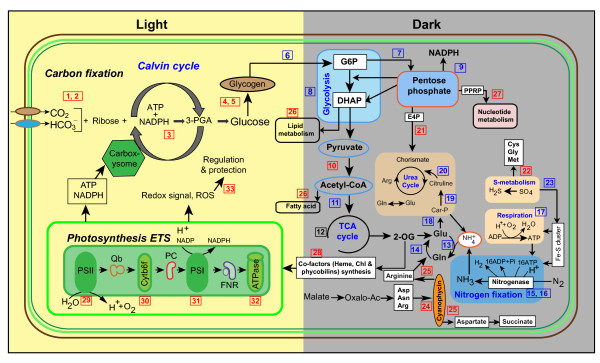
**Overview of the daily cellular metabolism in *Cyanothece *ATCC51142**. The biochemical pathways were predicted based on the current data and the available information in the literatures [11-13,16,39,52,60-62]. Each arrow shows the direction of the reaction. Boxed numbers denote individual or group of enzymes involved for various biochemical reactions, and color indicate whether the protein or majority of the proteins in the group have higher expression in the light (red) or in the dark (blue). The RIA values of these proteins can be found in Additional file 1, Table S2. 1, beta-carbonic anhydrase (IcfA1); 2, CO_2 _concentrating mechanism proteins (CcmM, CcmK2, CcmK4); 3, ribulose 1, 5-bisphosphate carboxylase oxygenase (RbcL); 4, glycogen synthase (GlgA1); 5, 1, 4-glucan debranching enzyme (GlgB1, GlgB2); 6, glycogen phosphorylase (GlgP1); 7, glucose-6-phosphate dehydrogenase (Zwf); 8, enzymes of glycolysis (Fba, Gap, Eno, Pgk, PykF1); 9, enzymes of pentose phosphate pathway (OpcA, TktA, TalA, Gnd, Pgl); 10, pyruvate dehydrogenase (PdhA, PdhC, IpdA); 11, citrate synthase (GltA); 12, enzymes of TCA cycle (AcnB, Icd, GabD, Mdh, SucC); 13, glutamine synthetase (GlnA); 14, glutamate synthase (GlsF); 15, nitrogenase (NifHDK); 16, nitrogenase co-factor biosynthesis (NifB, NifS); 17, respiratory enzymes (CobB1, PetH); 18, carbamoyl-phosphate synthase (CarB); 19, ornithine carbamoyltransferase (ArgF); 20, argininosuccinate synthetase (ArgG); 21, chorismate synthase (AroC); 22, S-adenosylmethionine synthetase (metK); 23, Fe-S cluster biosynthesis proteins (HesB), 24, cyanophycin synthetase; (CphA), 25, cyanophycinase (CphB); 26, enzymes of fatty acid/phospholipid metabolism; 27, enzymes of nucleotides metabolism; 28, co-factor biosynthesis enzymes; 29, PSII proteins; 30, cytochrome b6f complex (PetA, PetB, PetD); 31, PSI proteins; ATP synthase; 32, enzymes of stress and redox regulation. PSI, photosystem I; PSII, photosystem II; Qb, plastoquinone Qb; Cytb6f, cytochrome b6f complex; PC, plastocyanin; FNR, ferredoxin NADP reductase; ATPase, ATP synthase; 3-PGA, 3-phosphoglycerate; 2-OG, 2-oxoglutarate; Gln, glutamine; Glu, glutamate.

Synthesis of cyanophycin synthetase; CphA (cce_2237) (Figure 4B; bottom panel) was not coupled with the light-dark cycle, but cyanophycinase, CphB (cce_2236), which breaks down cyanophycin into arginine and aspartic acid, showed higher synthesis in the light cycle (Figure 4B, bottom panel). Arginine and aspartic acid can be utilized for the synthesis glutamate by combining with 2-OG and ammonia [45] (Figure 5) and might be utilized via the urea cycles as reported in *Synechocystis *PCC6803 [11] to obtain both alternative C and N sources..

Based on the quantitative information of new protein synthesis, we provided an overview of the temporal separation of cellular processes and metabolism in *Cyanothece *ATCC51142 during the light-dark cycle (Figure 5). Pathways were divided according to the diurnal period in which most of the proteins in a group are up-regulated. The timing was set based on the time course when the mean RIA of all members in a functional group (for example N_2_- fixation) for a given time-point was higher than the mean of all the time points. Different enzymes involved in multiple pathways linking carbon, organic acid and amino acid metabolism showed increased synthesis at different phases of the light-dark cycles suggesting a robust cellular programming in *Cyanothece *ATCC51142 to undergo diurnal cycles. Enzymes involved in carbohydrate metabolism (glycolysis, TCA cycle and oxidative PP cycle) usually showed higher synthesis in the dark and enzyme involved in CO_2 _fixation, stress and redox regulation, and co-factors synthesis showed higher synthesis in the light cycle (Figure 5). Importantly, enzymes involved in fatty acid and lipid metabolism including GlpK (cce_0973), FadD (cce_1133), FabD (cce_1583), PlsX (cce_1585) and GlpD (cce_2610) had a slight trend for increased synthesis either in the light cycle or during the transition from light to dark cycle. This might suggest that fatty acids and lipids are important sources of energy for many cellular functions. Additionally, photo-oxidative stress during active photosynthesis generates reactive oxygen species (ROS) and triggers redox signaling, apparently expressing enzymes of cellular protection and detoxification including Gor, Trx, Gst and SodB.

### Crosstalk between carbon metabolism and other intermediary pathways

The ability to link and quantify enzymes that link carbon, organic acid and amino acid metabolism is crucial to understanding metabolic regulation at multiple levels. Our analysis enables the linking of carbon metabolism with other central metabolism including lipids, fatty acids, amino acids (leucine and isoleucine), nucleic acids (purines and pyrimidines), heme and chlorophyll metabolism (Figure 5).

**Figure 6:**
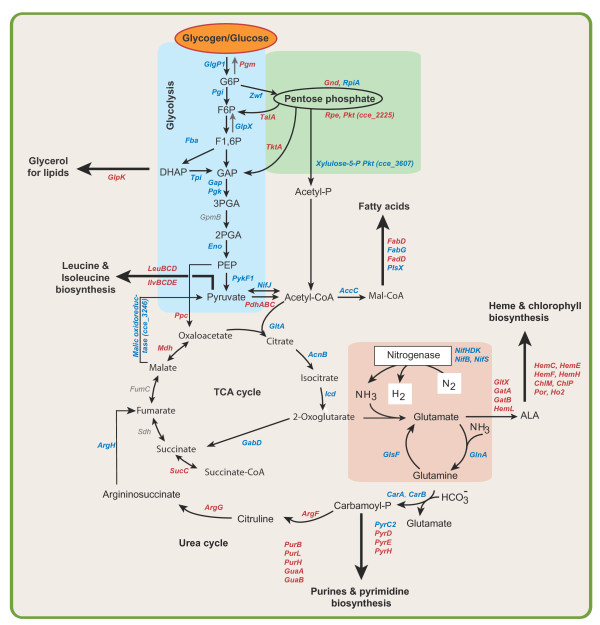
**Biochemical pathways and enzymes involved in carbon and nitrogen metabolism in *Cyanothece *ATCC51142**. Pathways were generated by mapping labeled proteins onto known metabolic pathways. Each arrow shows the direction of the reaction. Protein symbols in red indicate higher synthesis in the light or during the transition to dark. Protein symbols in blue indicate higher synthesis in the dark cycle or during the transition to light. Incomplete TCA cycle in cyanobacteria [42,63] provides 2-OG as a carbon skeleton for nitrogen assimilation through GS-GOGAT cycle converting 2-OG to glutamate which is utilized either for the biosynthesis of heme, chlorophyll, and phycobilin or purines and pyrimidines. The excess glutamate (or carbamoyl-P) is recycled through the urea cycle. The pathway also provides different intermediate metabolites as precursors for the biosynthesis of lipids (such as DHAP), fatty acids (Mal-CoA) and leucine and isoleucine (pyruvate and acetyl-CoA). The maximum levels of synthesis of these enzymes as determined by RIA are shown in parenthesis.

The heme biosynthetic pathway is fundamental for phycobilin and chlorophyll biosynthesis [46]. In parallel with phycobiliproteins, those involved in pigment biosynthesis also showed increased synthesis in the light cycle (Additional file 4, Figure S2) suggesting their role for chlorophyll biosynthesis and for the formation of PSI, PSII and cytochrome b6f complex [47]. Integration of this data suggests a novel pathway-crosstalk between central metabolism and the heme and chlorophyll biosynthetic pathway (Figure 6). Significant expression of GabD as mentioned before (Figure 4B, 6th panel) might possibly suggest the existence of an alternative route for the conversion of 2-OG to succinate (Figure 6). So, a portion of 2-OG might be channeled to produce succinate, and might be utilized as an alternative carbon source. Although, this pathway has been described in plants [48], to our knowledge, a similar short-cut shunt has not been described in cyanobacteria yet. Furthermore, significant up-regulation of PHK (cce_3607) during the dark cycle is striking (RIA = 0.62 at D2-1) (Figure 6), as this enzyme catalyzes the first step reaction to convert xylulose- 5P to pyruvate. In glycolytic-PP pathway, conversion of xylulose-5P to pyruvate generates an average of 1.67 moles of ATP per mole of xylulose-5P [49]. In contrast, conversion of xylulose-5P to pyruvate and acetyl-P by PHK pathway generates an average of 2 moles of ATP, and additional 1 mole of ATP is further produced by the conversion of acetyl-P to acetate. Therefore, the existence of a more energy efficient PHK pathway in cyanobacteria might be a cellular strategy and play an important role for improved cellular metabolism under limited condition.

### Chaperones, heat shock and stress responsive proteins

Quantitative profiles of chaperons and stress response proteins peaked at different stages of light-dark cycle (Additional file 4, Figure S2). While larger proportions showed relatively higher abundance in the light, others such as superoxide dismutase; SOD (cce_1620), glutathione S-transferase; Gst1 (cce_0174) and chaperonin 2; GroEL2 (cce_3330) showed higher abundance in the dark cycle. So, regulation of these proteins is presumably controlled by the metabolic state of the cell rather than the direct response to the light-dark cycle. ATP dependent Clp proteases also peaked at different time points (Additional file 4, Figure S2). Notably, regulatory subunits ClpC1 (cce_4247) and ClpB1 (cce_1941) showed higher synthesis in the dark but proteolytic subunits such as ClpP1 (cce_0641), ClpP2 (cce_4439), ClpP3 (cce_1911) and ClpP4 (cce_1910) showed higher synthesis in the light. Protein degradation plays a vital role in regulating cellular enzymes and regulatory proteins in cyanobacteria [50]. It is also responsible for targeting and efficient removal of damaged or otherwise functionally abnormal proteins such as oxygen sensitive nitrogenase before they reach levels toxic to metabolic process. This selective proteolysis in cyanobacteria is performed by different ATP dependent proteases, and their active synthesis suggests a robust targeted protein degradation mechanism, an agreement with the increased protein aggregation during the transition from dark to light cycle or the *vice-versa *and removal of aggregated proteins by different proteases. Additionally, carboxy terminal protease (cce_1372) and amino terminal peptidase, PepN (ce_0144) were also actively synthesized (Additional file 4, Figure S2). These peptidases are involved in proteolytic processing of proteins into mature forms [51], where carboxy terminal protease is responsible for cleavage of C-terminus residues, and PepN is involved in ATP-independent steps in the cleavage of amino terminus residues. As photosynthesis inherently produces strong oxidants and reductants, and utilizes multiple redox active intermediate electron transport chain, maintenance of redox balance is particularly challenging in photosynthetic microbes [52]. So, active synthesis of Clp proteases, chaperones and antioxidants suggest an extensive cellular response mechanism in *Cyanothece *ATCC51142 to deal with production of reactive oxygen species (ROS) and protein degradation during photosynthesis and N_2_-fixation.

## Conclusions

Overall, we have presented a dynamic proteomic profiling of *Cyanothece *ATCC51142 by using partial metabolic labeling and high mass accuracy LC-MS/MS analysis. Interrogation of the dynamic proteome over two consecutive diurnal cycles provided a unique picture of real-time protein synthesis in response to the light-dark cycle. Specifically, we identified 721 proteins actively synthesized over the time course, of which 425 proteins matched with the previously characterized cycling transcripts [12]. From this study, we were able to add qualitative and quantitative information at the proteome level, and the resulting view of the dynamic proteome enhanced our understanding of how cells modulate multiple metabolic pathways to switch molecular machinery daily to execute photosynthesis N_2_-fixation.

Nitrogenase enzymes were actively synthesized during the dark cycle. But, comparison with the mRNA level changes [12] revealed a temporal offset which can be related to the immediate cellular response to transcript levels and the time required to accumulate the translated nitrogenase enzymes. The nitrogenase proteins remained longer than the short-lived transcripts presumably due to a longer delay time for their degradation. Key proteins involved in C and N acquisition and assimilation were also actively synthesized at different phases of the light-dark cycles. Under nutrient limitation, *Cyanothece *ATCC51142 internally mobilizes alternative C and N sources using multiple pathways. Notably, our analysis revealed that 2-OG can be channeled for the production of glutamate via the GS-GOGAT pathway and for the production of succinate via TCA shunt (Figure 6). Furthermore, up-regulation of ArgG, ArgH and malate dehydrogenase (Mdh) might provide indirect evidence of the conversion of arginine and aspartic acid, released from cyanophycin into succinate to generate glutamate, ammonia and CO_2 _[11]. One of the interesting observations was the detection of active synthesis of proteins supporting the PHK pathway. As assimilation of C and N is an energy intensive process, requiring significant amounts of ATP and NADPH, utilization of this energy efficient PHK pathway by *Cyanothece *seems to be a cellular strategy for efficient metabolism under limited conditions. Our data also showed a robust cellular mechanism for targeted degradation of proteins involving several proteases.

Despite extensive sampling and pre-fractionation, protein level expressions of several cycling transcripts were not observed, likely due to their low abundance [12]. However, by only focusing on actively synthesized proteins, we find a majority of these overlap with cycling transcripts. The remaining 296 proteins represent an interesting case however, where these proteins are under active transcription and translation based upon the detected metabolic label, but which do not fall within the "cycling" transcripts. It could be that these proteins are simply constitutively expressed at low levels regardless of cycle, or that they require such high degradation/turnover which precludes any observable cyclic behavior at the gene or protein level. Accordingly, the output of the current study has substantial biological implications and further in-depth analysis with more temporally and spatially restricted samples will allow deeper exploration of the *Cyanothece *ATCC51142 proteome and almost certainly result in the discovery of additional cellular features. Furthermore, a significant number of labeled proteins were of unknown functions, and functional studies of these new and previously uncharacterized proteins will provide valuable insights into utilizing *Cyanothece *ATCC51142 as a viable large-scale component of carbon sequestration or renewable energy production. To summarize, the combination of sub-fractionation techniques with state-of-the art metabolic labeling and high accuracy LC-MS/MS analysis provided a comprehensive overview of the highly dynamic temporal proteome of *Cyanothece *ATCC51142 in between the light-dark cycles with useful information on the actual flux-state of proteins in a biological sample, which has direct implicates to cellular functions.

## Methods

### Culture conditions and labeling experiment

Stock cultures of *Cyanothece *ATCC51142 were maintained in ASP_2 _medium [3] with NaNO_3 _under conditions of continuous light at 50 μmol photons.m^-2^.s^-1 ^[12]. For the experiment, *Cyanothece *ATCC51142 cells were grown in ASP_2 _medium [3] without NaNO_3 _at 30°C under 12 h light/12 h dark cycles and 50 μmol photons.m^-2^.s^-1 ^of light for 7 days (chlorophyll concentration of 0.97 μg/ml of cells) before adding 20 mg/l of [^13^C_6 _^15^N_7_]-leucine (U-^13^C_6_, 98%; ^15^N_7_, 98%; Cambridge Isotope Laboratories Inc., MA). Cultures were grown for a 12 h period in the light to allow for sufficient isotope incorporation before first sampling at 1 h after the onset of the dark period. Samples were collected at 4 h intervals. A total of 13 samples were collected for analysis with the last sample collected at 1 h into the third diurnal cycle (D3-1; Figure 1).

### Sample preparation

Soluble and insoluble fractions from total cell extracts were prepared as described before [11,16]. Protein concentrations were determined using the bicinchoninic acid (BCA) assay (Pierce Chemical Co., Rockford, IL). Soluble proteins were denatured and reduced in 8 M urea and 5 mM tributylphosphine (Sigma-Aldrich) at 37°C for 60 min. The insoluble fraction was treated identically, except for the addition of 1% CHAPS for 45 min prior to digestion. All samples were then diluted 5 times in 25 mM ammonium bicarbonate prior to tryptic digestion using sequencing-grade modified porcine trypsin (Promega, Madison, WI) at a 1:50 (w/w) trypsin-to-protein ratio for 5 h at 37°C. Peptides were desalted using a 1 ml C-18 SPE column (Supelco, St. Louis, MO) [53]. Samples were separated using strong cation exchange chromatography (SCX) with a PolySulfoethyl A, 200 mm × 2.1 mm, 5 μM, 300-Å column and a 10 mm × 2.1 mm guard column (PolyLC, Inc., Columbia, MD) at a flow rate of 0.2 ml/min as previously described [54,55]. The peptides were re-suspended in 900 μl of mobile phase A (0.2% FA in water), and separated on an Agilent 1100 system (Agilent, Palo Alto, CA) equipped with a quaternary pump, degasser, diode array detector, Peltier-cooled auto-sampler and fraction collector (both set at 4°C). A total of 24 fractions were collected for each sample (soluble or insoluble protein) and pooled in such a way as to make 6 fractions for soluble protein and 4 fractions for insoluble protein for subsequent MS analysis.

### Reversed Phase Liquid Chromatography (LC) Separation and Tandem MS (MS/MS) Analysis

Samples were analyzed using high throughput hybrid LC-MS/MS of the trypsin-digested proteome samples [16]. Six (6) μl of the tryptic peptides containing 3 μg of the total peptides from each SCX fraction out of 60 μl per fraction were analyzed by capillary LC (150 μm i.d. × 360 μm o.d. × 65 cm capillary; Polymicro Technologies Inc., Phoenix, AZ) under constant pressure of 5000 psi coupled with a Finnigan LTQ Iontrap Mass Spectrometer (ThermoFinnigan, San Jose, CA) and an electrospray ionization source manufactured in-house as described in [56]. The LC column was prepared by slurry-packing 3-μm Jupiter C_18 _bonded particles (Phenomenex, Torrence, CA) into a 65-cm long, 75-μm inner diameter fused silica capillary (Polymicro Technologies, Phoenix, AZ). After loading peptides onto the column, peptides were separated for a 100 min run at a flow rate of ~1.8 μl/min using the following settings: 100% mobile phase solvent A (0.2% formic acid in water) for 20 min, followed by a linear gradient from 0 to 70% of solvent B (0.2% formic acid in 100% acetonitrile) over 80 min before reverting to 100% solvent A. Each full MS scan (m/z 400-2000) was followed by collision-induced MS/MS spectra (normalized collision energy setting of 35%) for the 10 most abundant ions per duty cycle selected for fragmentation. The dynamic exclusion window was set to 30 s, the heated capillary was maintained at 200°C, and the ESI voltage was held at 2.2 kV.

### Data analysis and relative isotope abundance (RIA) measurement

The MS/MS data were processed using MaxQuant software [57] version 1.1.1.2. The MaxQuant package integrates a suite of software packages for peak list generation, labelled peptide pairs, SILAC- and XIC-based quantitation, false discovery rates [58], and peptide identification based on Mascot [42] search [18]. The step by step details of this procedure can be found in reference [57]. Briefly, MaxQuant executes data analysis in 4 sequential steps. First step performs 'pre-processing and quantitation' to identify all the peptide pairs without knowing the identity, and then performs Mascot search in the second step. Mascot search was performed using version 2.2.04 against the concatenated forward and reverse versions of the *Cyanothece *ATCC51142 protein sequence databases (updated on June 21, 2010) downloaded from NCBI http://www.ncbi.nlm.nih.gov/protein that contained 5304 protein sequences. Mascot search parameters included: enzyme trypsin, one missed cleavage, oxidation of methionine and ^13^C^15^N-leucine (7 Da) as variable modifications. The maximum allowed mass error tolerances for MS and MS/MS scans were 5 ppm and 0.5 Da, respectively. Peptides with ≥6 amino acid residues were only allowed, and proteins that had at least two peptides with ion scores > 20 were considered for identification and quantitation. A target decoy database approach using forward-reverse sequences and contaminants was used to determine false discovery rate (FDR) and FDR threshold was set to the default values of 1% for both the peptides and the proteins and posterior error probability (PEP) of 0.5%. The identified and quantified peptide pairs are then visualized into two-dimensional (2D) and three-dimensional (3D) peak formats. 2D peaks are generated based on local maxima of the intensity as a function of m/z, centroid and assembled into 3D peak hills over the m/z-retention time plane. Two peaks in the neighbouring scans are connected if their centroid m/z positions differ by < 7 ppm. At least two centroids should match to form a 3D peak and the centroids that cannot be matched to centroids in the two previous or the two next scans are discarded. This removes a major fraction of the noise in form of peaks not reproducible in time. Additionally, intensity profiles of the pairs should have a sufficient overlap in retention time by requiring them to have a uncentered Pearson correlation of > 0.6. Then, the intensity profiles are smoothed over retention time applying a window mean filter of ± 1 scan width and checked for local minima. Intensities of heavy and light peptides thus generated were used to determine the Relative Isotope Abundances (RIA) described previously [21,23,24] as:

RIA_t _= I_H_/(I_H _+ I_L_), where I_H _and I_L _represent the intensities of heavy and light peptides, respectively at time t. RIA measures the relative abundance of the label peptides (I_H_) to the total peptide pool (I_H_+I_L_) in the cell. So, rapidly synthesized proteins accumulate label at a faster rate than the low turnover proteins, and proportionally have higher RIA values. The protein RIA at any particular time point was calculated based on the averaged peptides RIAs per protein. As labelled peptides contain the mixture of both fully and partially labelled peptides (in case of multiple Leu containing peptides), both peptide mixtures were used to calculate the RIA. In each time point, a labelled protein was identified by the pair of stable isotope labelled and the corresponding unlabeled variant, and accordingly, under the experimental set-up, most of the proteins would increase in the label through the time of the experiment, but at different rates. In LC-MS, the partition of the peptide ion current between the heavy and light pairs provided the rate at which that protein was newly synthesized. The Mascot output files were combined with time-course information, metabolic functions and other relevant information using Microsoft Office Access 2007 and then loaded into Data Analysis Tool Extension (DAnTE) version 1.2 [59] for clustering and downstream analysis. DAnTE uses abundance (expression ratios) data or the spectral count data, and allows users to apply many analysis algorithms for data normalization, peptides rollup into proteins, data plotting and statistical analysis. The RIA values of individual peptides were log2 transformed, normalized to correct variations in labelling intensities of peptides, rolled up into proteins (Reference peptide based roll up; RRollup) and performed hierarchical clustering and visualized as heat maps. Labelled proteins were excluded from the list if they were identified by a single peptide pair and observed only at one time-point out of the 13 time points. But, proteins identified by a single peptide pair were accepted if that peptide was unique to that protein and identified in at least 2 out of the 13 time points. Proteins identified only at one time point were accepted if they contain ≥ 2 labelled peptides pairs. Proteins identified by shared peptides were accepted if they included at least one unique peptide pair.

A robust approach to determine both cycling *Cyanothece *transcripts and cycling proteins from near identical experiments have been previously reported [12,16], which was used in the current study for a baseline comparison of the current results.

## Abbreviation used

SILAC: stable isotope labeling by amino acid in cell culture; XIC: extracted ion chromatogram; MS: mass spectrometry; LC-MS/MS: liquid chromatography tandem mass spectrometry; BCA: bicinchoninic acid; CID: collision induced dissociation; RIA: relative isotope abundance; CHAPS: 3-[(3-cholamidopropyl)dimethylammonio]-2-hydroxy-1-propanesulfonate; FDR: false discovery rate; NCBI: National Center for Biotechnology Information; SCX: strong cation exchange chromatography; SPE: solid phase extraction; iTRAQ: isobaric tag for relative and absolute quantification; 2-OG: 2-oxoglurarate; Rubisco: 1: 5-bisphosphate carboxylase-oxygenase; GS: glutamine synthetase; GOGAT: ferredoxin dependent glutamate synthase; PHK: phosphoketolase; PSI: photosystem I; PSII: photosystem II; PQ: plastoquinone; PC: plastocyanine; OEC: oxygen evolving complex; PBS: phycobilisome; ALA: 5-aminolevulinnate; ROS: reactive oxygen species.

## Competing interests

The authors declare that they have no competing interests.

## Authors' contributions

UKA, JS, JMJ, DWK, RDS and HBK conceived and designed the experiment. UKA, JMJ, RK, RJM and MEM performed analysis and interpretation of the data, UKA wrote the manuscript. JS, JMJ, HBP and RDS edited the manuscript. MAG performed sample preparation. All authors read and approved the final manuscript.

## Supplementary Material

Additional file 1 **Table S2**. List of all the 721 labeled proteins with their ORF, gene identifier, functional annotations, protein descriptions, and the log2 transformed RIA values across different time points. RIA was calculated as the ratio of the heavy peptide intensity (IH) to the sum of heavy and light peptides intensity (I_H_+I_L_); (RIA = I_H_/(I_H_+I_L_)). The consecutive diurnal cycles are labeled as D1, D2, and D3 for the dark and L1 and L2 for the light. The extensions (-1), (-5) and (-9) indicate 1, 5, 9 hrs into the dark and the light cycles. The values are given as the normalized average log2 ratios of the labeled peptides (listed in the Additional file 1, Table S1) corresponding to each proteins. ** ORFs are labeled according to the deposited genome sequence in GenBank (accession nos. CP000806 - CP000811). Click here for file

Additional file 2 **Table S1**. List of all the 2837 labeled peptides with their ORF, functional annotations, protein descriptions, and the actual RIA values across different time points. RIA was calculated as mentioned before, and are the actual values which were used for given log2 transformation and rollup into proteins as listed in the Additional file 2, Table S2. ** ORFs are labeled according to the deposited genome sequence in GenBank (accession nos. CP000806 - CP000811). Click here for file

Additional file 3 **Figure S1**. (A), Distribution of the labeled peptides/proteins at different time points. (B), Venn diagram showing the comparison of the current labeled proteomic data with the previous transcriptomic data [12] and the label-free quantitative proteomic data [16]. Click here for file

Additional file 4 **Figure S2**. Heatmaps showing light-dark induced changes in protein abundances of different functional groups over time. The complete lists of all the proteins with their RIA values are shown in Additional file 2, Table S2. Psaf, photosystem II stability/assembly factor; D-X5P/D-F6P, putative D-xylose-5-phosphate/D-fructose 6-phosphate phosphoketolase; Trx, thioredoxin. Click here for file
