# Supplementary figures and images for: Dynamic proteomic profiling of a unicellular cyanobacterium Cyanothece ATCC51142 across light-dark diurnal cycles
Source: BMC Syst Biol. 2011 Dec 1;5:194. doi: 10.1186/1752-0509-5-194 (PMC3261843; doi:10.1186/1752-0509-5-194)

**(A)**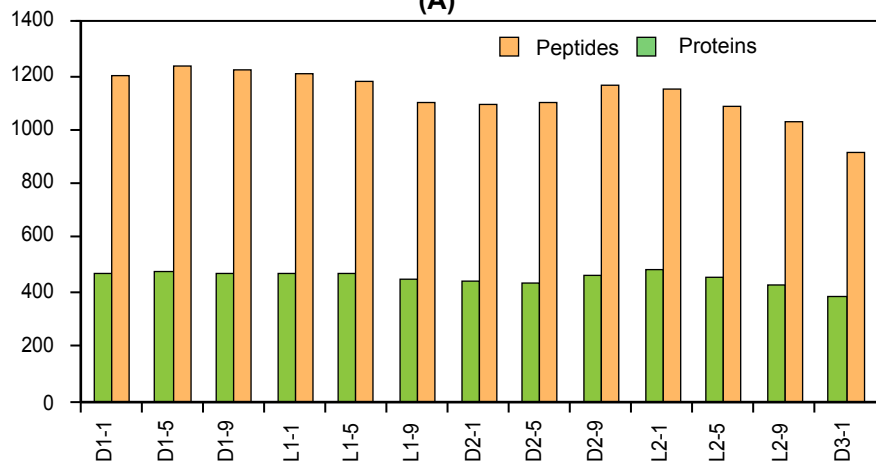**(B)**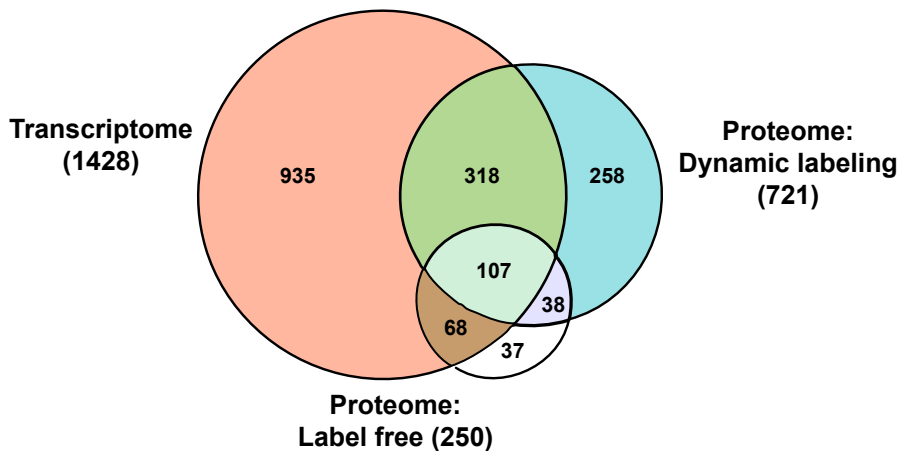

Supplement: Additional file 3 — Figure S1. (A), Distribution of the labeled peptides/proteins at different time points. (B), Venn diagram showing the comparison of the current labeled proteomic data with the previous transcriptomic data [12] and the label-free quantitative proteomic data [16]. [file 1752-0509-5-194-S3.PDF]

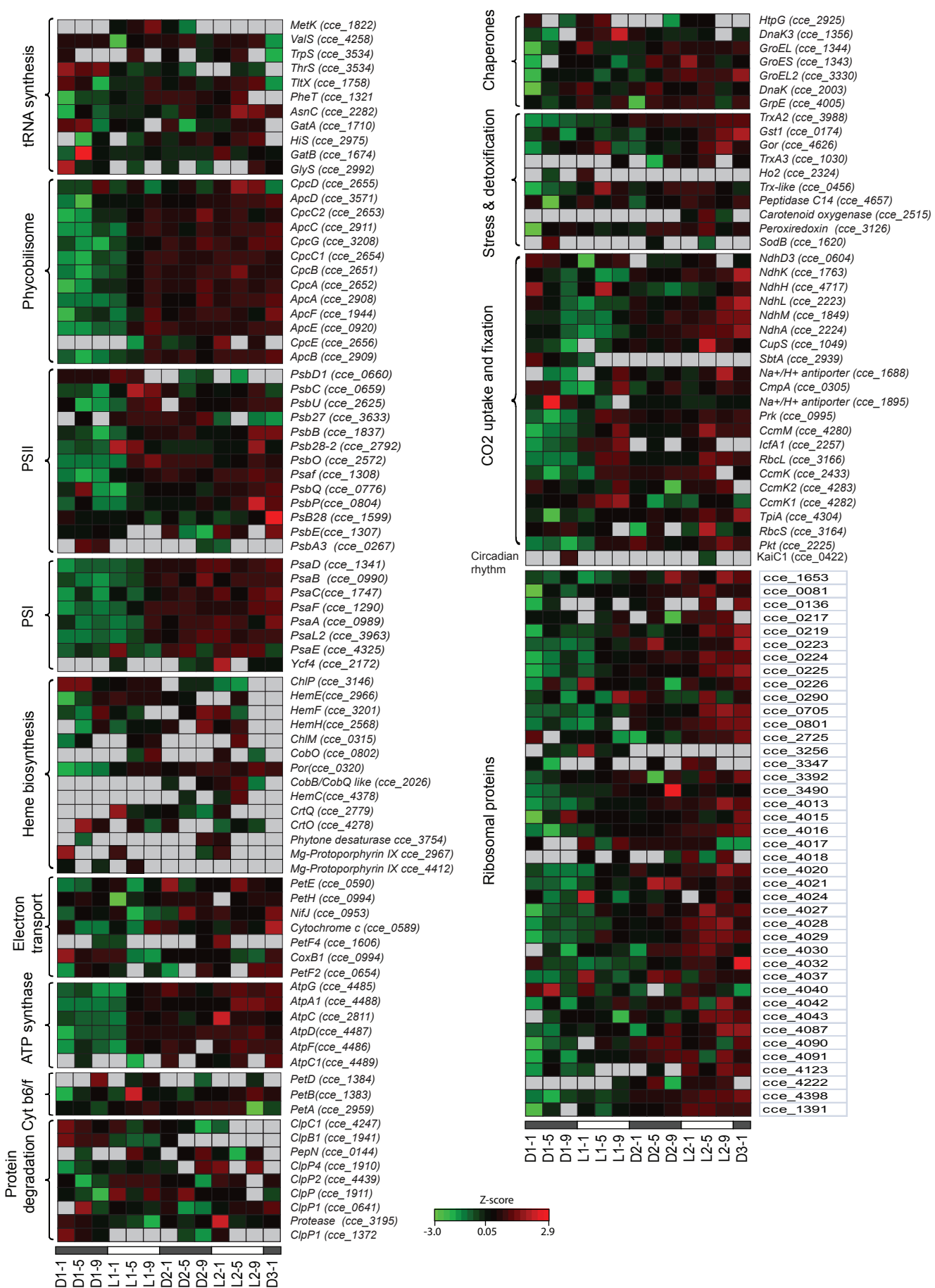

Supplement: Additional file 4 — Figure S2. Heatmaps showing light-dark induced changes in protein abundances of different functional groups over time. The complete lists of all the proteins with their RIA values are shown in Additional file 2, Table S2. Psaf, photosystem II stability/assembly factor; D-X5P/D-F6P, putative D-xylose-5-phosphate/D-fructose 6-phosphate phosphoketolase; Trx, thioredoxin. [file 1752-0509-5-194-S4.PDF]
